# P078 Polymyalgia rheumatica masquerading as infective spondylodiscitis: PET-CT prevents unnecessary spinal biopsy

**DOI:** 10.1093/rap/rkag113.108

**Published:** 2026-11-03

**Authors:** Kriti Sharma, Urwah Ahmed, Marvi Iftikhar, Azeem Ahmed

**Affiliations:** Oxford University Hospitals NHS Foundation Trust, Oxford, United Kingdom; Oxford University Hospitals NHS Foundation Trust, Oxford, United Kingdom; Oxford University Hospitals NHS Foundation Trust, Oxford, United Kingdom; Oxford University Hospitals NHS Foundation Trust, Oxford, United Kingdom

## Abstract

**Introduction:**

Polymyalgia rheumatica (PMR) classically presents with symmetrical shoulder and pelvic girdle stiffness reflecting bursitis and tenosynovitis around the shoulders and hips, responding rapidly to low-dose steroids. Atypical presentations with spinal and sacroiliac inflammation on MRI can closely mimic infective spondylodiscitis, prompting antibiotic treatment and consideration of invasive spinal biopsy. We describe a 68-year-old woman with osteoarthritis, osteoporotic vertebral fractures and longstanding back and girdle pain, who presented acutely with fever, markedly elevated inflammatory markers and MRI changes suggestive of spondylodiscitis. PET-CT demonstrated a pattern characteristic of PMR, without evidence of infection, malignancy or large-vessel vasculitis, avoiding spinal biopsy.

**Case description:**

A 68-year-old woman with osteoarthritis, osteoporosis (fragility vertebral fractures 2012 and 2017), hypertension and longstanding intermittent back, hip and shoulder pain (previously investigated in 2017 shoulder ultrasound showing tenosynovitis and supraspinatus tear) presented with a three-week history of fever, fatigue, severe left hip and shoulder pain, stiffness, weakness, and swollen left ankle and knee, associated with reduced mobility.

She had recent foreign travel and a sore throat prior to symptom onset.

Examination showed reduced proximal power in the left upper and lower limbs (attributed to pain), bilateral positive FABER’s test, cervical spine and sacroiliac joint tenderness, and mild left ankle synovitis, without rash or other extra-articular findings.

Blood tests showed markedly elevated inflammatory markers (CRP 283mg/L, ESR 130mm/hr, ferritin 600µg/L) with neutrophilia; creatine kinase was normal, and procalcitonin remained low throughout. Urine dipstick was positive but culture negative, and blood cultures were negative. ECHO requested was rejected. Axial MRI showed multilevel vertebral bone marrow oedema and sacroiliitis, reported as suggestive of spondylodiscitis. She was empirically treated with antibiotics for presumed infection. Given persistently high inflammatory markers, spinal biopsy was considered but deferred considering negative infection screen pending further specialist radiological opinion. A review comparing with imaging from 2017 and 2023 now favoured non-radiographic axial spondyloarthropathy.

To further characterise the disease pattern non-invasively and exclude infection, malignancy and large-vessel vasculitis (LVV) in one whole-body study, PET-CT was performed, showing marked bilateral periarticular shoulder avidity and mild hip periarticular avidity consistent with PMR, without evidence of LVV, infection or malignancy and thus avoiding spinal biopsy.

She was commenced on prednisolone 30mg daily with rapid improvement within days, alongside bone protection (bisphosphonate, calcium and vitamin D). Serial bloods confirmed CRP normalisation, and rheumatoid factor, anti-CCP and HLA-B27 were negative, excluding alternative seropositive arthritis. She remains on a gradual steroid taper with rheumatology follow-up.

**Discussion:**

This case illustrates two diagnostically challenging aspects of PMR: its capacity to mimic infective spinal disease, and the value of PET-CT in resolving diagnostic uncertainty and avoiding invasive investigation. PMR classically presents with girdle pain and stiffness responding rapidly to low-dose steroids, but atypical presentations with high inflammatory markers, fever, or spinal and sacroiliac involvement can closely resemble infection, leading to delayed treatment and unnecessary investigation, like recently reported cases of atypical PMR mimicking infection. In our patient, marked elevation of CRP, ferritin and ESR, together with MRI bone marrow oedema initially interpreted as sacroiliitis, prompted treatment for presumed spondylodiscitis with empirical antibiotics, despite negative cultures and low procalcitonin, and consideration of spinal biopsy.

FDG-PET CT findings in PMR and axial spondyloarthritis can overlap, so distinguishing the two on imaging alone is not always straightforward; meta-analyses report a sensitivity of around 85% and specificity around 80% for PET-CT in diagnosing PMR, with typical patterns including bilateral shoulder, hip and peri-articular bursal uptake. In this case, PET-CT findings were considered more characteristic of PMR than active axial spondyloarthritis, showing marked bilateral periarticular shoulder avidity with only mild hip involvement and no enthesopathic or large-vessel avidity, while also excluding infection and malignancy in a single whole-body study.

This case demonstrates the importance of reviewing serial imaging alongside clinical trajectory and highlights the dual diagnostic value of PET-CT: identifying a pattern characteristic of PMR while simultaneously excluding infection, malignancy and large-vessel vasculitis, thereby avoiding an unnecessary and potentially higher-risk spinal biopsy.

Response to corticosteroids, together with normalisation of inflammatory markers and negative rheumatoid factor, anti-CCP and HLA-B27, further supported the diagnosis and excluded coexisting inflammatory arthritis.

This case reinforces the principle that atypical or diagnostically uncertain presentations of PMR warrant whole-body imaging to exclude mimics before committing to invasive investigation or antimicrobial therapy.

**Key learning points:**

• PMR can present atypically with high fevers, very high inflammatory markers, and axial or sacroiliac involvement, closely mimicking infective spondylodiscitis or spondyloarthropathy, and should remain a differential even when initial findings suggest infection.

• Comparing new imaging with prior studies can help distinguish acute infective change from longstanding inflammatory disease, avoiding unnecessary invasive investigations such as spinal biopsy.

• PET-CT can be a valuable tool in atypical PMR, both to characterise the distribution of periarticular and girdle inflammation typical of PMR and to exclude large-vessel vasculitis,i nfection and malignancy in a single investigation, potentially avoiding unnecessary invasive investigations such as spinal biopsy.

• A rapid, dramatic clinical response to low-dose corticosteroids remains a strong supportive feature of PMR and should prompt diagnostic reconsideration when initial treatment for a presumed infective or malignant cause has been ineffective.

• Baseline and follow-up screening for alternative inflammatory arthritides, including rheumatoid factor, anti-CCP and HLA-B27, is useful in atypical presentations to exclude overlapping or alternative diagnoses.

• Pre-existing osteoporosis and vertebral fragility fractures should prompt early, proactive bone protection when initiating long-term glucocorticoid therapy for PMR.

